# Lung Hydatidosis Unveiled: A Multisystem Mirage of Pathological Rarity

**DOI:** 10.7759/cureus.52819

**Published:** 2024-01-23

**Authors:** Mihir Patil, Pankaj Gharde, Kavyanjali Reddy

**Affiliations:** 1 General Surgery, Jawaharlal Nehru Medical College, Datta Meghe Institute of Higher Education and Research, Wardha, IND

**Keywords:** albendazole, hydatid cyst, zoonotic, multiorgan hydatid disease, echinococcosis, hydatid disease

## Abstract

Lung hydatidosis is a zoonotic infection, primarily caused by *Echinococcus *sp., and has a significant role in the economy and public health. Canines are the predominant hosts of these parasitic tapeworms. Lungs are the most commonly infected organ after the liver. Hepatic pulmonary blood circulation is mainly reported to transport infection to the lungs. The prevalence of hydatid disease has increased over the past decade. In addition, patients with multi-organ involvement of hydatid cysts have been reported in different parts of the world. Hydatidosis can remain asymptomatic for years after infection in some conditions, especially in cases of splenic hydatidosis. Chest radiography and computed tomography findings can be used to confirm the diagnosis of hydatid disease. Hydatid disease is, in general, managed by pharmacological therapy, but if multi-organ involvement is observed, surgery along with medical management is required. Long-term follow-up is recommended in such cases to check the recurrence of the disease.

## Introduction

Hydatid disease is a zoonotic disease commonly caused by *Echinococcus granulosus* and *Echinococcus multilocularis* in humans. It is commonly observed in individuals associated with agriculture and animal keeping, with a prevalence of 0.5% to 4%, although prevalence rates may be higher in areas having close associations with livestock [1,2]. Although hydatid disease can affect any organ, the liver and lungs are most commonly affected. This is because they can act as a filter, trapping Echinococcal larvae during their life cycle in the circulatory system. Entrapped larvae in the liver potentially cause hydatid cysts in the liver and the larvae escaping the microcirculatory vessels reach the lungs, infecting them [1]. Hydatid cysts can occur in different organs such as the liver, spleen, lungs, brain, peritoneum, thyroid, and mesentery or may spread through systemic dissemination, which might go unnoticed without any clinical symptoms [3]. Hydatid cysts of *E. granulosus* form as unilocular fluid-filled bladders in the internal organs of humans and other intermediate hosts, primarily the liver and lungs. Available molecular investigations have reported nine different genotypes of *E. granulosus* based on its mitochondrial DNA sequence [4]. The two additional species that are also reported to cause infection in humans are *Echinococcus vogeli* and *Echinococcus​*​​​​​​* oligarthrus*, which induce polycystic echinococcosis in rare instances. Currently, ultrasound imaging and computed tomography are helpful imaging modalities aiding the diagnosis of hydatid disease [5].

## Case presentation

**Figure 1:**
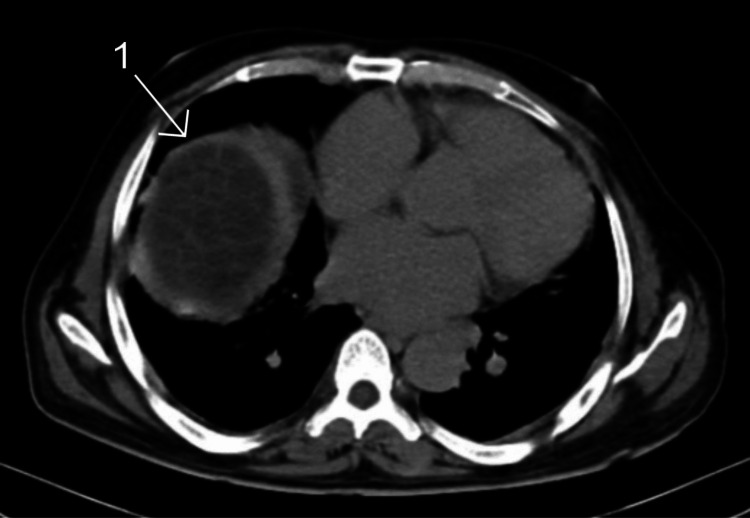
Computed tomography report showing a hydatid cyst in the lung. 1: A hydatid cyst in the right lung containing multiple daughter cysts.

A 52-year-old male presented with dull, aching pain in the right hypochondriac region that radiated toward the left hypochondrium for four months. The patient also had breathlessness, cough, and intermittent fever, which was associated with nausea, vomiting, and weight loss. The patient was a shepherd by occupation and had regular contact with livestock and dogs. The patient had been on medical treatment for jaundice for the past five months, but there was no previous documentation available with him. The patient had a poorly built physique and was moderately nourished in appearance. The patient exhibited pallor and mild icterus, with no signs of cyanosis, clubbing, or lymphadenopathy. Vital signs upon admission were as follows: a pulse rate of 122/min, blood pressure of 100/70 mmHg, and oxygen saturation levels of 90% on room air. Physical examination revealed a soft and tender abdomen on the right. The left hypochondrium revealed no guarding, rigidity, distension, or palpable mass. Hepatosplenomegaly was observed on further examination. Auscultation revealed decreased air entry over the right side of the chest and normal air entry over the left side of the chest. The computed tomography (CT) report was suggestive of a hydatid cyst in the right lung (Figure 1).

**Figure 2:**
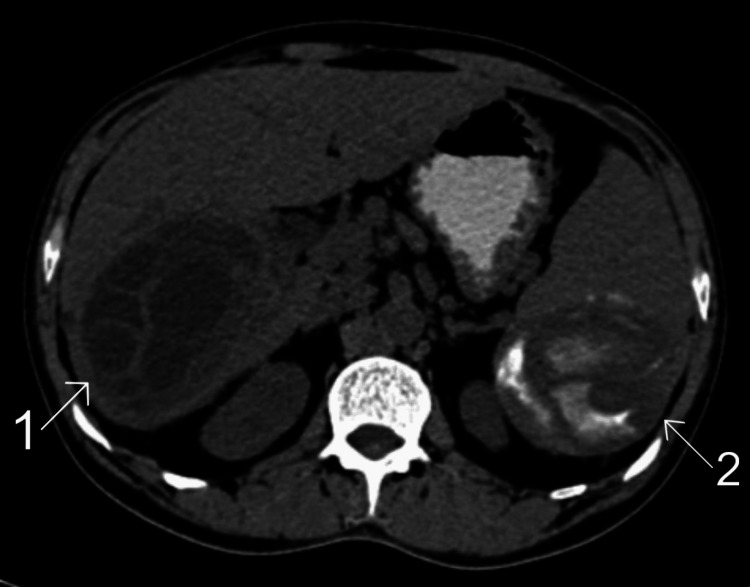
Computed tomography of the abdomen in the patient. 1, hydatid cyst in the liver; 2, hydatid cyst in the spleen

**Figure 3:**
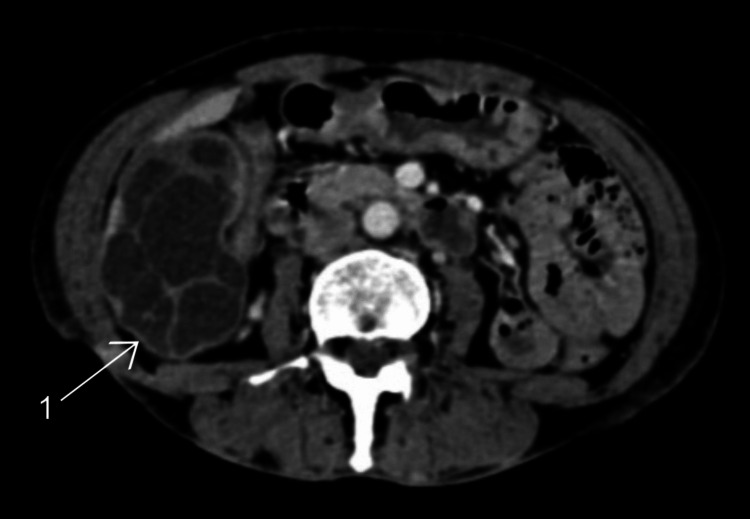
Computed tomography image of the abdomen. 1, hydatid cyst in the liver

**Figure 4:**
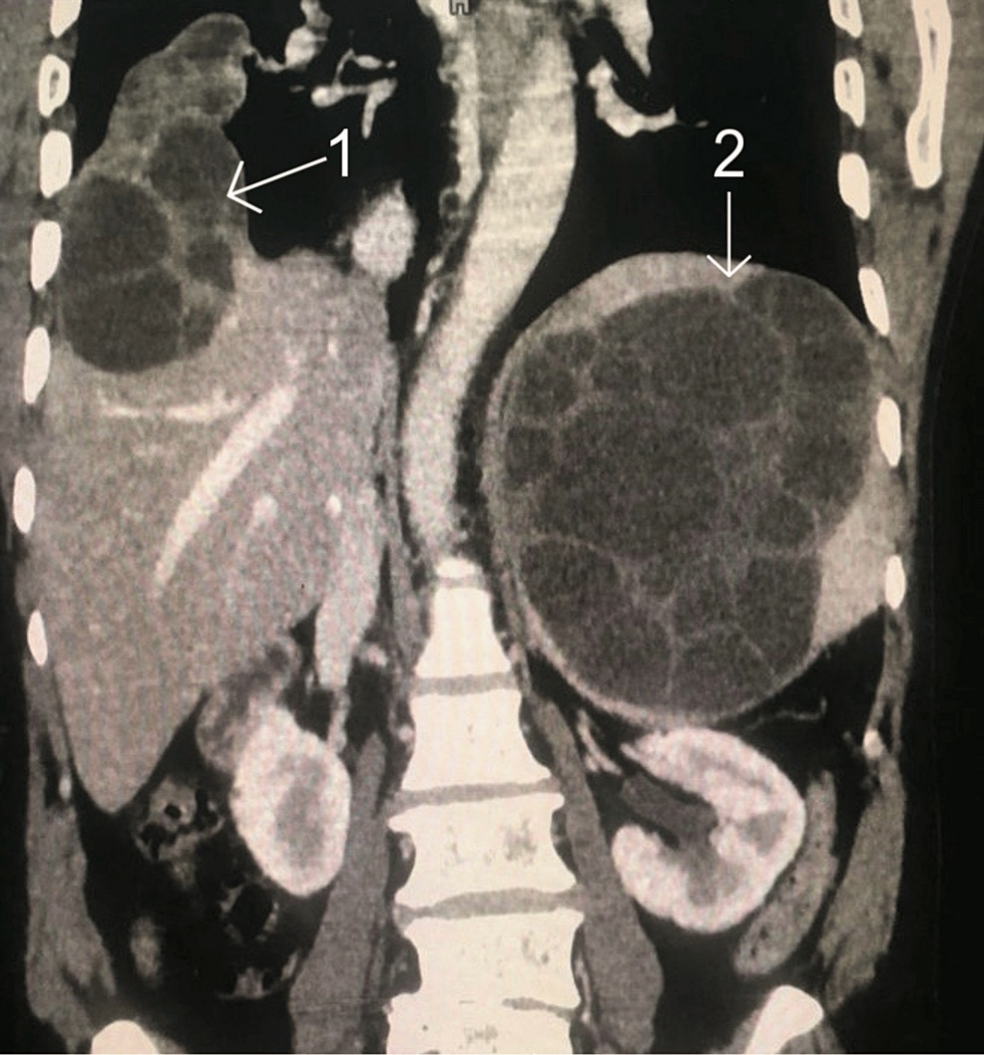
Computed tomography image showing a view of hydatid cysts presence in the liver and spleen. 1, hydatid cyst in the liver; 2, hydatid cyst in the spleen

The abdominal CT indicated the presence of large, well-defined multi-loculated cystic lesions in the right lung, along with multi-loculated lesions in the segments of the liver. These liver lesions showed septations and tiny foci of calcifications, suggesting hydatid cysts in the liver, spleen, and lung (Figures 2-4).

A lesion causing a mass effect, manifested as the compression of intrahepatic biliary radicals and prominence of inter-hepatic biliary radicals along with the common bile duct, was indicative of hepatomegaly, with the liver measuring 19.2 cm. The spleen size was noted to be 14 cm, displaying a similarly large, well-defined cystic lesion in the splenic parenchyma with multiple foci and the presence of daughter cysts. These findings lead to the conclusion that the reports suggest hydatid cysts of the spleen. Based on these findings, hydatid cysts of the liver, lung, and spleen were confirmed. The patients' laboratory investigation profile is presented in Table 1.

**Table 1:** Lab investigation profile of the patient. LFT, liver function test

Blood parameter	Value	Normal range
Hemoglobin	7.1 g/dL	12-15.8 g/dL
White blood cells	24,300/μL	4,500-11,000/μL
Platelet	2.25 lacs/μL	1.5-4.5 lacs/μL
Urea	48 mg/dL	5-20 mg/dL
Creatinine	2.0 mg/dL	0.6-1.2 mg/dL
Erythrocyte sedimentation rate	126 mm/hour	<50 mm/hour
C-reactive protein	170 mg/dL	<0.9 mg/dL
LFT		
Alkaline phosphatase	230 U/L	40-112 U/L
Aspartate amino transferase	102 U/L	10-40 U/L
Alanine aminotransferase	88 U/L	7-41 U/L
Total protein	6.1 g/dL	6.0-8.5 g/dL
Albumin	2.6 g/dL	3.5-5.5 g/dL
Total bilirubin	3.8 mg/dL	0.3-1.3 mg/dL
Conjugated bilirubin	3.1 mg/dL	0.0-0.35 mg/dL
Unconjugated bilirubin	0.7 mg/dL	0.2-0.65 mg/dL

Case management

**Figure 5:**
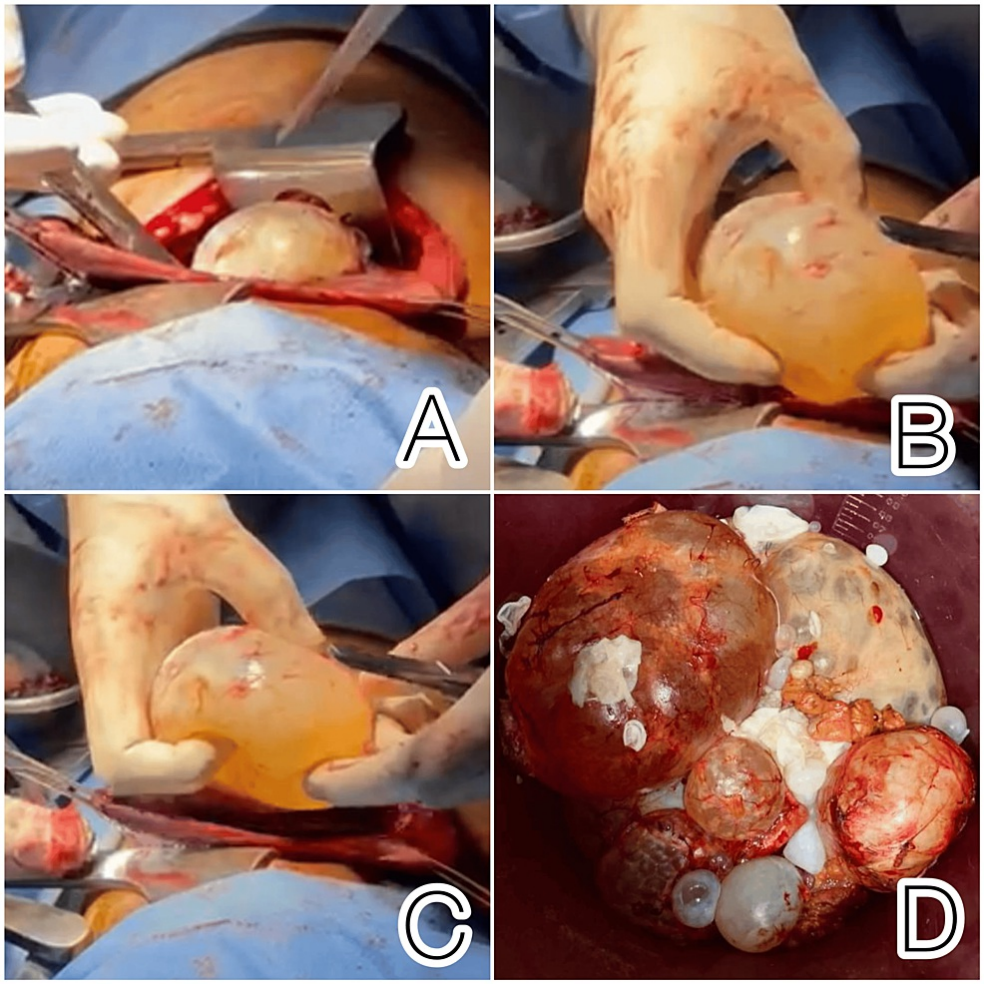
Intraoperative images of hydatid cysts extraction and extracted cysts. (A)-(C) Right thoracotomy and in toto extraction of hydatid cysts from the right lung; (D) extracted cysts

Preoperatively, the patient was transfused with two units of packed red blood cells (PRBCs), one unit of whole blood, and three units of platelets. Increased creatinine levels were managed through hydration over the course of several days, and urinary output was monitored based on the guidance provided by the nephrologists. The patient was administered antibiotics for two weeks before surgery, and prophylactic therapy with albendazole was started immediately after the diagnosis. Surgery was planned after obtaining fitness clearance from the anesthesia and medicine teams. The operative management involved a right thoracotomy and an approach to extract the hydatid cyst of the lung without any spillage, a procedure that was successfully completed. A right subcostal incision was made to extract the hydatid cyst in the liver. All daughter cysts were extracted with due precautions, with no spillage in the peritoneal cavity. A left subcostal incision was made to extract hydatid cysts of the spleen, and marsupialization of splenic hydatid cysts was completed (Figure 5).

The contents inside the cavity were scraped off, and marsupialization of the splenic hydatid cyst was performed. This was followed by washing the peritoneal cavity with a cetrimide solution and subsequent cleaning with hypertonic saline. Hemostasis was achieved. The abdominal layers were closed, and the skin was sutured. The postoperative period was uneventful, and the patient had no complaints other than mild pain at the incision site. Albendazole treatment was continued. The samples were sent for histopathology, and the report confirmed a hydatid cyst of the liver and spleen.

## Discussion

Research reports have mentioned that patients are asymptomatic for years post-infection [1]. Hydatid disease has been reported to commonly affect organs such as the lungs, liver, and spleen, but it is not limited to these and might infect other organs such as the kidneys, brain, heart, and bones [4,5]. These infections have been reported in different parts of the world, majorly prevalent in people associated with farming and cattle rearing. When associated with the spleen, it has been reported mainly in four major forms: hydatid cysts, epidermoid cysts, pancreatic pseudocysts, and post-trauma pseudocysts. Hydatid cysts can exhibit various clinical presentations, and their radiological appearance can also vary. Radiological diagnostic methods can be utilized for the differentiation of different types of cysts into active, inactive, or transitional, which can be crucial to deciding the course of its clinical management based on its biological activity [6]. However, patients with hydatid cysts involving multiple organs are rare and warrant more reporting. This would contribute to a better understanding of the disease, its prevalence, awareness, and management strategies. There are very few reports, primarily from the last decade, documenting multi-organ involvement in hydatid disease, both in children and adults. We have compiled cases similar to ours from articles with available open-access data (Table 2).

**Table 2:** Patients reported for multi-organ hydatid disease.

Multi-organ hydatid disease reported (number of cases/article)	Location	Year	Reference
1	South Africa	2022	Ntatamala et al. [7]
3	India	2022	Mandal et al. [8]
36	Turkey	2020	Tercan et al. [9]
1	Afghanistan	2020	Shabbir et al. [10]
1	India	2021	Behera et al. [11]
1	Turkey	2016	Tekin et al. [12]
1	Turkey	2008	Olmez et al. [13]

Hydatid disease is considered to have originated in rural areas, but in recent times, there have been reports of cases from the cities as well [14]. According to Pukar and Pukar, the management of hydatid cysts should be tailored to each patient's specific needs [15]. Although a few original concepts have been proposed, the etiology and pathophysiology of this disease are yet to be understood. Albendazole is found to be an effective adjuvant therapy for treating hydatid cysts, and patients who receive albendazole therapy have lower chances of recurrence [1,16]. Albendazole alone may be used to treat cysts that are less than 5 cm in size. Puncture, aspiration, injection, and re-aspiration (PAIR), a percutaneous drainage procedure, is one of the most recently created alternatives to surgery. The danger of allergy and peritoneal seeding is minimized by using PAIR to enter the cyst cavity through the organ parenchyma while guided by ultrasound and computed tomography scan. Based on available research reports, the PAIR approach seems to be a safe method for treating cysts with a diameter of 50 mm, especially for patients who refuse surgical therapy or have an intolerably high risk for anesthesia. The PAIR approach may be used alone or in conjunction with albendazole to treat cysts larger than 5 cm [17]. A standardized and consistent management approach is needed for lung hydatidosis based on the patient's clinical condition, host factors, and surgical risk, which can vary on individual case presentations. Research evidence suggests that a strong partnership between infectious disease specialists and surgeons can create practical guidelines, simplifying the decision-making process for clinicians and enhancing optimal practices [18]. Ozgokce et al. documented a case involving a three-year-old child with massive hydatid cysts in both the lungs and liver. They noted that the coexistence of such large cysts in both liver and lungs is rare in children of this age group. In such cases, immediate surgical intervention is recommended to treat hydatid cysts for a better outcome [19]. Although hydatid disease is an endemic concern in a few countries of the world, there is a rise in the number of cases with an equal prevalence in children and adults. Hence, there is an urgent need to address further research on its preventive strategies and management approaches.

## Conclusions

Hydatid disease, if diagnosed on time, can lead to better clinical outcomes and fewer post-surgical complications. The case highlights successful surgical management of lung, liver, and splenic hydatid cyst extraction. Multiple cysts from the lung and liver were extracted using a proper surgical approach, and marsupialization of splenic hydatid cysts was done. Preoperative transfusion, hydration, and antibiotics were administered, followed by meticulous cyst(s) removal. Postoperatively, the patient experienced mild pain and was continued treatment with albendazole. Future research is needed to guide the implementation of improved preventive measures, increasing awareness, and enhanced medical record-keeping to optimize patient care in endemic regions. Although surgery remains the most effective and conclusive treatment for hydatid disease, a multidisciplinary approach is essential for managing such cases effectively and decreasing the recurrence with long-term follow-ups.
